# Broad-scale lake expansion and flooding inundates essential wood bison habitat

**DOI:** 10.1038/ncomms14510

**Published:** 2017-02-23

**Authors:** Jennifer B. Korosi, Joshua R. Thienpont, Michael F. J. Pisaric, Peter deMontigny, Joelle T. Perreault, Jamylynn McDonald, Myrna J. Simpson, Terry Armstrong, Steven V. Kokelj, John P. Smol, Jules M. Blais

**Affiliations:** 1 Department of Biology, University of Ottawa, Ottawa, Ontario, Canada K1N6N5; 2 Department of Geography, York University, Toronto, 4700 Keele Street, Ontario, Canada M3J 1P3; 3 Department of Geography, Brock University, St. Catharines, Ontario, Canada L2S 3A1; 4 Department of Geography and Environmental Studies, Carleton University, Ottawa, Ontario, Canada K1S 5B6; 5 Department of Physical and Environmental Sciences, University of Toronto Scarborough, Toronto, Ontario, Canada M1C 1A4; 6 Environment and Natural Resources, Government of the Northwest Territories, Fort Smith, Northwest Territories, Canada X0E 0P0; 7 Northwest Territories Geological Survey, Government of the Northwest Territories, Yellowknife, Northwest Territories, Canada X1A 2L9; 8 Paleoecological Environmental Assessment and Research Lab (PEARL), Department of Biology, Queen's University, Kingston, Ontario, Canada K7L 3N6

## Abstract

Understanding the interaction between the response of a complex ecosystem to climate change and the protection of vulnerable wildlife species is essential for conservation efforts. In the Northwest Territories (Canada), the recent movement of the Mackenzie wood bison herd (*Bison bison athabascae*) out of their designated territory has been postulated as a response to the loss of essential habitat following regional lake expansion. We show that the proportion of this landscape occupied by water doubled since 1986 and the timing of lake expansion corresponds to bison movements out of the Mackenzie Bison Sanctuary. Historical reconstructions using proxy data in dated sediment cores show that the scale of recent lake expansion is unmatched over at least the last several hundred years. We conclude that recent lake expansion represents a fundamental alteration of the structure and function of this ecosystem and its use by Mackenzie wood bison, in response to climate change.

## Abstract (web summary)

Climate change can affect lake water level and nearby landscape. Korosi and colleagues show recent expansion of shallow lakes in the Canadian Northwest Territories is flooding critical habitat for the wood bison, and demonstrate the trickle-down effect of climate change on ecosystem functioning.

The development and implementation of strategies for biological conservation related to the anticipated effects of climate change on ecosystems is a global priority^1^ and requires an understanding of how broad-scale climate changes interact with biodiversity and the conservation of vulnerable species of wildlife. In Arctic and subarctic ecosystems, climate change has led to hydrological changes that have important consequences for species conservation, including the drying of lakes and ponds in some areas and rapid lake expansion in others^2^. The predominant mechanisms behind lake level decreases have been well documented, such as the drying of High Arctic ponds as a result of increased evaporation^3^ or permafrost thaw-induced lake drainage^4^. The mechanisms driving large increases in lake surface area are less well understood. This represents a key knowledge gap, as widespread lake expansion may lead to the loss of essential terrestrial habitat and the flooding of terrestrial soils can stimulate the production of toxic methylmercury^5^. A dramatic example of regional lake expansion having an impact on the conservation of a species at risk is occurring in the Great Slave Plains and Lowlands ecoregions of the southern Northwest Territories (Canada), where the Mackenzie wood bison (*Bison bison athabascae* Rhoads 1897) population is found.

Wood bison are the largest land mammals in North America. Historically, the wood bison range extended over most of the boreal forest region of western North America, but over-hunting, in conjunction with long-term (∼5,000 years) changes in habitat, led to the near-extinction of this species by the end of the nineteenth century^6^. Currently, as a consequence of re-introduction and conservation efforts, herds of wood bison can be found in Alberta, Manitoba, British Columbia, the Yukon and the southern Northwest Territories of Canada, as well as in Alaska and Siberia. In Canada, wood bison are currently designated as a threatened species under Canada's Species at Risk Act because of a number of factors. These include habitat loss, small population effects, introgressive hybridization and management controls to limit population sizes to reduce the potential transmission of reportable cattle diseases to non-infected populations^7^. The Mackenzie Bison Sanctuary was established in 1963 in the Great Slave Plains and Lowlands ecoregion of the southern Northwest Territories. It is home to a genetically pure herd of wood bison that have not genetically mixed with the more abundant and closely related plains bison (*Bison bison bison*)^8^. The Mackenzie herd is also one of the largest wood bison herds not endemically infected by tuberculosis and brucellosis^9^. For these reasons, it is imperative from a conservation perspective to protect the Mackenzie herd. The conservation of suitable foraging habitat has been identified as an essential element of wood bison recovery efforts^10^.

Observational evidence indicates that many shallow lakes, ponds and wetlands in the Mackenzie Bison Sanctuary have dramatically increased in surface area over the recent past, a trend opposite to the decreases in lake area that have been documented across much of northern Canada^2^. Lake expansion has flooded large areas of sedge-grass meadows previously identified as optimal bison forage habitat^10^. The observed expansion has largely involved the refilling of dry, relatively flat lacustrine basins, indicating that lake areas had been larger in the past. The flat terrain, low hydraulic gradients, presence of discontinuous permafrost and fine-grained glaciolacustrine deposits characteristic of the Great Slave Plains and Lowlands impede drainage and may contribute to the responsiveness of this landscape to fluctuations in water balance. A cyclical pattern of lake expansion and retraction may actually be an important contributor to the suitability of this region as bison habitat, because periodic flooding events would prevent the encroachment of tree cover and promote the maintenance of sedge-grass meadows. The hydrological responsiveness of this landscape, however, also makes it challenging to predict how climate change disrupts natural expansion and retraction cycles.

In this study, we investigate the mechanisms and historical context behind recent lake expansion in the Mackenzie Bison Sanctuary using remotely sensed images and lake sediment cores. We explore the climatic variables that best explain changes in lake area over the recent past as inferred from the remote sensing record, changes in the spatial distribution of the Mackenzie wood bison population following recent lake expansion and the potential cyclicity of these events over the last several hundred years using lake sediment records as proxy archives of long-term, decadal trends in lake surface area. Specifically, our aim is to determine whether current increases in lake area are within the range of natural variability of these ecosystems, or represent an overall net positive change in water balance as a likely consequence of anthropogenic climate change. We show that the proportion of the landscape occupied by water nearly doubled between 1986 and 2011, and, in response, the Mackenzie Bison herd are now avoiding their former core habitat. Inter-annual variability in lake size between 1986 and 2011 is significantly correlated with climate teleconnection patterns and local temperature/precipitation records, but water levels remain high despite the drought-like conditions that have persisted in the region since 2013. Historical reconstructions using proxy data archived in dated sediment cores show the scale of recent lake expansion is unmatched over the last several hundred years.

## Results

### Recent changes in lake area from 1986 to 2011

**Figure 1:**
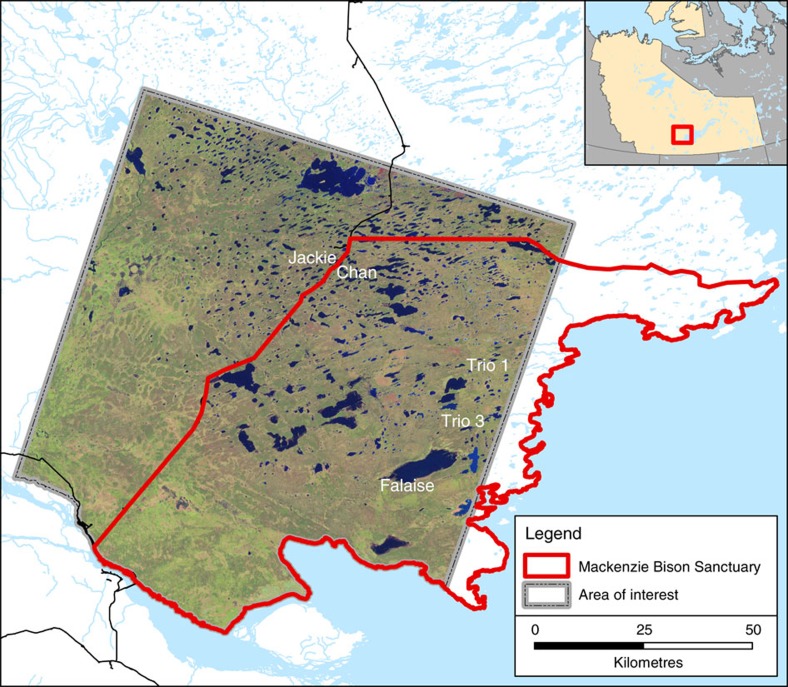
Map of the study region. Map showing the location of the Mackenzie Bison Sanctuary (in red) and the area of the Great Slave Plains and Lowlands ecoregion analysed in this study with Landsat imagery outlined in grey (Landsat imagery obtained from United States Geological Survey Earth Explorer; see Methods for details). Geospatial data for basemap from Natural Resources Canada CanVec+ database. Locations of the five study lakes are marked. Inset shows the location of the Northwest Territories in Canada.

**Figure 2:**
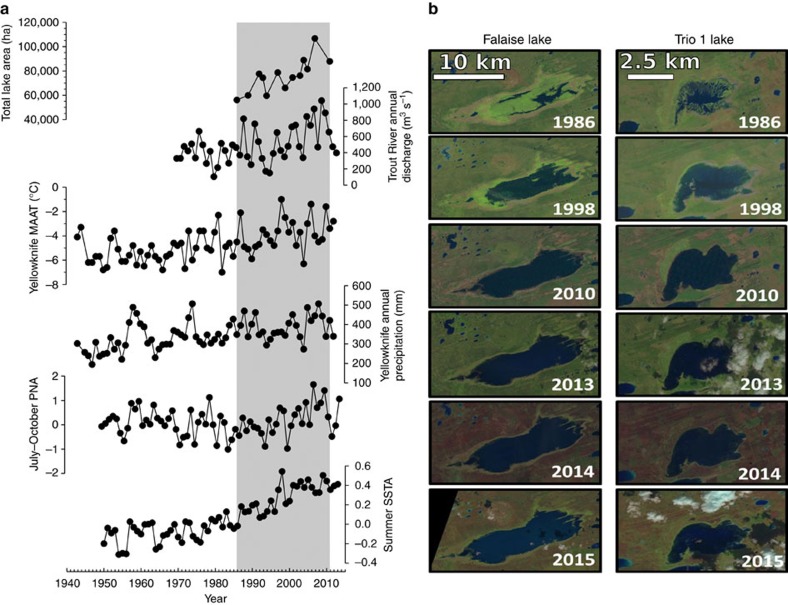
Climate and lake area trends in the Mackenzie Bison Sanctuary (NT). (**a**) Temporal trends in overall lake area (ha) from 1986 to 2011 in the Great Slave Plains and Lowlands estimated from Landsat imagery; annual discharge from the nearby Trout River; mean annual air temperature (MAAT) at Yellowknife; annual precipitation at Yellowknife; July–October PNA teleconnection pattern; and global summer (June-August) SSTA based on HadSST2 data set since 1940. (**b**) Fall (12 Aug 12–13 October) Landsat images collected in 1986, 1998, 2010 and 2013–2015 from Falaise Lake and Trio 1 Lake, showing no notable reductions in lake area since 2012, despite the dry conditions that have persisted in the region over the last few years.

Fall (August–October) Landsat 5 Thematic Mapper images available since 1986 were obtained for a large (∼10,000 km^2^) region encompassing most of the Mackenzie Bison Sanctuary and a roughly equal area outside the sanctuary to the north and west of Highway 3 (Fig. 1). Although considerable inter-annual variability was evident, analysis of Landsat imagery revealed that the proportion of the landscape occupied by water has nearly doubled over the Landsat record, from ∼561.2 km^2^ (5.7% of the total study area) in 1986 to a maximum of 1,067.1 km^2^ (11%) in 2007 (Fig. 2a). In 2011, ∼9% of the landscape was occupied by water (Fig. 2a). Despite the general trend of increasing lake area, the magnitude and timing of lake expansion has been heterogeneous across the landscape. In recognition of this, we selected five lakes for detailed study (Fig. 1 and Supplementary Table 1).

Falaise Lake (61.47642°N, 116.15280°W) is the largest lake contained within the Mackenzie Bison Sanctuary. The surface area of Falaise Lake increased markedly from 6.1 km^2^ in 1986 to 43.4 km^2^ in 1992, after which lake area declined again to 21.1 km^2^ in 1997 (Fig. 2b). A second period of expansion in Falaise Lake occurred from 1997 to 2011, increasing to 56.4 km^2^ in size by 2011, a total increase of 824% since 1986. The ‘Trio' lakes, a series of three lakes centrally located in the Mackenzie Bison Sanctuary (unofficially named Trio 1, Trio 2 and Trio 3), are a classic example of the heterogeneous nature of recent lake expansion. Trio 1 (61.64026°N, 116.05184°W) experienced a similar pattern of expansion as Falaise Lake, exhibiting an initial expansion phase between 1986 and 1992 (from 1.8 to 9.7 km^2^), followed by a period of retraction in lake size, and a second phase of expansion after 2000. The surface area of Trio 1 in 2011 was 10.4 km^2^, a total increase of 462% (Fig. 2b). Trio 3 (61.59762°N, 116.07063°W) has not exhibited any major changes in surface area since 1986 (surface area ∼3.0 km^2^). The remaining two study lakes are smaller systems, each located immediately adjacent to Highway 3. Chan Lake (61.89079°N, 116.54170°W) exhibited a 51% increase in surface area over the recent past (from 0.5 to 0.7 km^2^), relatively small compared with the scale of expansion observed for many other lakes in the region. Jackie Lake (61.89678°N, 116.55987°W; unofficial name) exhibited one phase of expansion, unlike the two-phase expansion pattern observed for Falaise Lake and Trio 1 Lake, increasing from 0.6 to 1.5 km^2^ (312% increase) between 2001 and 2011.

### Changes in wood bison distribution

**Figure 3:**
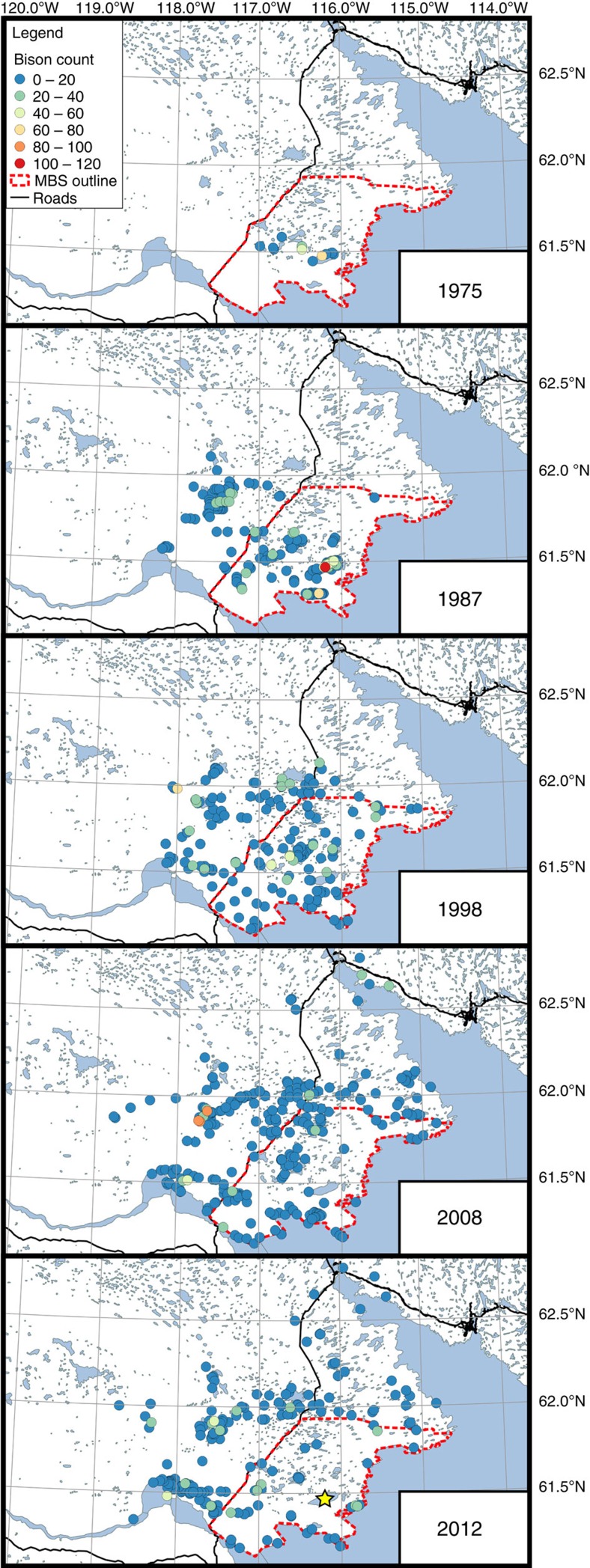
Spatial distribution of the Mackenzie wood bison herd since 1975. Map comparing the number and spatial distribution of bison observed in the Mackenzie Bison Sanctuary and surrounding areas based on aerial population surveys conducted in 1975, 1987, 1998, 2008 and 2012. Yellow star denotes the location of Falaise Lake. Geospatial data for basemap from Natural Resources Canada CanVec+ database.

Systematic aerial population surveys have been conducted at regular intervals since 1975 to monitor the movements of the Mackenzie wood bison herd. In 1975, the herd was located entirely within the borders of the Mackenzie Bison Sanctuary, including more than a hundred bison congregated around Falaise Lake (Fig. 3). Increasing population numbers after 1975 led to the range expansion of the herd. High numbers of bison were still observed within the Mackenzie Bison Sanctuary, including at Falaise Lake, but bison were also found west of Highway 3 (Fig. 3). By 2008, the number of bison recorded within the core area of the Mackenzie Bison Sanctuary declined substantially and bison were more populous along the margins of the sanctuary, congregating alongside Highway 3, or to the west and north of the sanctuary (Fig. 3). By 2012, no bison were observed at Falaise Lake (Fig. 3).

### Climatic drivers of variability in lake area

Climate records from Fort Providence are sparse and the entire period of Landsat coverage corresponded to an absence of measurements at Fort Providence (Supplementary Fig. 1). Annual temperature trends at Fort Providence for the period with records (1943–1962 and 1973–1982) were found to be significantly and positively correlated to the longer temperature records from the nearby cities of Yellowknife and Hay River (Supplementary Table 2). A significant, albeit weaker correlation also existed between Yellowknife and Fort Providence for annual and seasonal (except winter) precipitation (Supplementary Table 2). Therefore, to investigate the climatic drivers of inter-annual variability in lake surface area since 1986, Landsat-inferred regional lake surface area was compared against seasonal/annual temperature and precipitation records from Yellowknife, major teleconnection patterns (for example, El Nino Southern Oscillation, Pacific Decadal Oscillation, Arctic Oscillation and the Pacific/North American (PNA) pattern), sea surface temperature anomaly (SSTA) and discharge records from the Trout and Cameron rivers, using individual Spearman's rank correlations and generalized additive models.

The July to October PNA pattern was the variable that best correlated to lake area (*r*_s_=0.70, *P*<0.001; Fig. 2a). No other teleconnection was significantly correlated. In addition, summer SSTA (*r*_s_=0.56, *P*=0.04) and annual discharge from the Trout River (*r*_s_=0.59, *P*=0.03) were positively correlated with water level (Fig. 2a). The Trout River is located ∼300 km west of the Mackenzie Bison Sanctuary and receives waters from creeks and lands between its origin at Trout Lake and its termination at the Mackenzie River. Generalized models were generated for individual and multiple explanatory variables as well, including all of the local climate and hydrological data, as well as teleconnections. The model that best predicted lake area included annual discharge from both the Cameron and Trout rivers as predictors (Supplementary Table 3). Similar to the results from the Spearman's rank correlations above, July to October PNA pattern, Trout River annual discharge and global summer SSTA resulted in models that strongly predicted changes in regional lake area (Supplementary Table 3). Annual temperature combined with the Trout River annual discharge, as well as annual temperature combined with annual precipitation, also resulted in models that predicted lake area well (Supplementary Table 3).

### Historical reconstructions of lake expansion events

**Figure 4:**
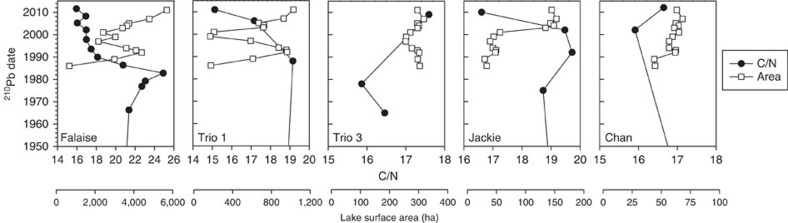
Decreasing C/N in sediment cores tracks recent lake expansion. Recent (since 1950) atomic organic C/N ratio in the sediment (black circles) and Landsat-derived lake surface area estimates (white squares) for the five study lakes.

Elemental and stable isotope analyses of carbon and nitrogen were investigated as potential proxy records for inferring past lake expansion events in our five study lakes (Falaise, Trio 1, Trio 3, Jackie and Chan). A decrease in organic carbon to nitrogen (C/N) elemental ratios clearly tracked the timing of recent lake expansion in Falaise Lake, Jackie Lake and Trio 1 Lake (Fig. 4), and downcore C/N was significantly negatively correlated to Landsat-derived lake surface area for Falaise Lake and Jackie Lake (*r*_s_=−0.83, *P*=0.02 and *r*_s_=−0.90, *P*<0.001, respectively). No major down-core changes in C/N were observed in Chan Lake or Trio 3 Lake, neither of which have experienced recent lake expansion (Fig. 4). The lowest C/N values for all expanded lakes were recorded in the upper sediments (Supplementary Fig. 2). The stable isotopic composition of C and N was not a good proxy record for inferring recent lake expansion in these systems (Supplementary Note 1).

**Figure 5:**
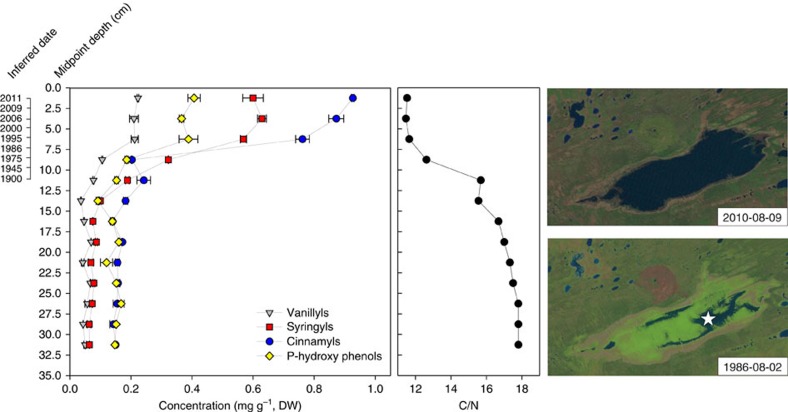
Lignin-derived phenols track the recent expansion of Falaise Lake. Stratigraphic profile of temporal changes in the concentration of four major lignin-derived phenol groups and atomic organic C/N ratio, in a sediment core from Falaise Lake. It is noteworthy that the timing of the decrease in C/N differed from the duplicate sediment core obtained from Falaise Lake shown in Fig. 4 (see Supplementary Note 2). Landsat images of Falaise Lake captured in 1986 and 2010 are shown on the right. The star on the 1986 image represents the sediment core retrieval location for the two sediment cores obtained from Falaise Lake in March 2012 and March 2013. Mean±error bar for s.d. of duplicate samples.

Lignin-derived phenols were analysed in an independently collected and dated sediment core from Falaise Lake, as an additional proxy for inferring past expansion events. Lignin-derived phenols track the influx of terrestrial organic matter to lake sediments, as lignin has no significant aquatic sources^11^. Lignin-derived phenols clearly recorded the post-1986 expansion of Falaise Lake (Fig. 5). We observed an increase in each of the four major lignin-derived phenol groups (vanillyls, syringyls, cinnamyls and p-hydroxy phenols) and, although a gradual increase in vanillyls, syringyls and p-hydroxy phenols began post-1850, further increases occurred after ∼1990 (Fig. 5). The largest magnitude, post-expansion increase (286%) was observed for the cinnamyls (Fig. 5).

## Discussion

Our palaeolimnological analyses provide evidence that, despite the susceptibility of these shallow systems to short-term variability in lake area, recent observed lake expansion represents an overall net positive change in water balance, probably as a consequence of anthropogenic climate change. Recent expansion in Falaise Lake, Trio 1 Lake and Jackie Lake was clearly reflected by a decrease in C/N, which was not observed in lakes that have not undergone recent expansion. The carbon and nitrogen composition of organic matter in lake sediments is derived from both autochthonous (in-lake) and allochthonous (for example, terrestrial) sources. Cellulose-rich, protein-poor vascular plant tissue generally has a C/N >20 and fresh algal-derived organic matter that is high in nitrogen-rich proteins and lipids usually exhibits C/N values between 4 and 10 (ref. 12). The decrease in C/N following expansion is probably tracking an increase in available habitat for phytoplankton production, as the surface area and volume of water in the lake basins increased. The lowest C/N values for all expanded lakes were recorded in the upper sediments, providing evidence that current lake expansion in the region is unmatched over at least the last several hundred years.

C/N in lake sediments can be susceptible to diagenesis^13^ and can also be influenced by a variety of limnological processes independent of lake expansion, such as increased algal production from a longer ice-free season (Supplementary Note 2). Therefore, we conducted a separate palaeolimnological analysis of lignin-derived phenols in a duplicate, independently dated sediment core from Falaise Lake, to confirm our interpretation that recent lake expansion is unmatched over the recent past in the Mackenzie Bison Sanctuary. Lignin is produced exclusively by vascular plants, with minimal aquatic sources^11^, and the inundation of terrestrial soils following lake expansion is expected to result in a pulse of lignin-derived phenols to lake sediments, providing an unambiguous marker of lake expansion events. This approach has been used successfully to track terrestrial soil inundation following reservoir impoundment^14^,^15^, an analogous situation to the natural expansion occurring in the Great Slave Plains and Lowlands. Lignin-derived phenols in the sediments of Falaise Lake clearly recorded the post-1986 expansion of Falaise Lake, especially the cinnamyls. Cinnamyls are abundant in non-woody angiosperms such as grasses and sedges^11^, which are the most common vegetation type along the margins of Falaise Lake, and the preferred food sources of wood bison^16^. At no other point in the sediment record did we observe an increase in cinnamyl concentrations that would indicate a previous expansion event of sustained duration had occurred in Falaise Lake over the last several hundred years.

Two independent palaeolimnological reconstructions of historical lake level dynamics (using C/N and lignin-derived phenols) independently confirmed that post-1986 lake expansion in the Mackenzie Bison Sanctuary represents a net positive change in water balance outside of the range of natural variability, at least over the last several hundred years. This, in combination with significant positive correlations between 1986 and 2011 lake area and the July–October PNA pattern (a positive PNA phase is associated with warmer, wetter conditions in Yellowknife^17^) and summer SSTA implicate recent climate change as a probable cause. When combined, annual temperature and precipitation were moderately good predictors of inter-annual variability in lake area; however, despite persistent dry conditions in the southern Northwest Territories since 2012, water levels remain high. This suggests that the climatic drivers behind recent lake expansion are complex and not solely related to precipitation and evaporative balance. The Mackenzie Bison Sanctuary is underlain by discontinuous permafrost, but the rapid rate of enlargement, involving basin refilling in many cases, precludes thawing permafrost as a predominant mechanism of these observed changes, although permafrost thaw-induced changes to groundwater inputs into the expanding lakes may be a contributing factor. Further study is ongoing to resolve the underlying mechanisms of lake expansion in this region.

The dramatic landscape flooding that has occurred in the Great Slave Plains and Lowlands since the mid-1980s is the net consequence of complex hydrological processes under a changing climate and represents a fundamental shift in ecosystem structure that may permanently alter habitat use in the Mackenzie Bison Sanctuary by the Mackenzie wood bison herd. Both Falaise Lake and Trio 1 Lake were identified as excellent habitat for bison because of the extensive sedge meadows available for grazing in the basin^8^,^10^ and hundreds of bison were counted at Falaise Lake in 1987 (Fig. 3). Thus, the observed expansion of water coverage on the landscape is disproportionately flooding essential bison habitat by inundating sedge meadows that were prevalent in the previously dry lacustrine basins, causing the Mackenzie herd to abandon the former core of its range within the sanctuary in search of forage.

Bison movements, caused by habitat loss and alteration, have led to higher numbers of bison being struck by vehicles in recent years^18^ and increase the potential for interactions with diseased bison herds located further south (such as in Wood Buffalo National Park^19^), migratory caribou or other competitors they have not encountered before. The conservation challenges presented by recent bison movements are also occurring in a multiple-stressor context. In 2012, a large anthrax outbreak severely depleted the population size of the Mackenzie bison herd^20^. In 2014, much of the Mackenzie Bison Sanctuary was burned by one of the biggest fire complexes ever documented in the region, further altering wood bison habitat. The cumulative effects of these stressors provide a powerful demonstration of how anthropogenic climate change profoundly impacts the conservation of ecologically important wildlife species.

## Methods

The Government of the Northwest Territories has monitored the Mackenzie wood bison herd since the establishment of the Mackenzie Bison Sanctuary in 1963. Bison population estimates were obtained from aerial surveys conducted biannually from 1964 to 2000 (ref. 8), and in 2008 (ref. 8) and in 2012.

Landsat 5 Thematic Mapper images were identified and utilized for all analyses and accessed via the United States Geological Survey Earth Explorer website (http://earthexplorer.usgs.gov/, last accessed on 9 September 2015). Images were selected from within a consistent time period (12 August to 13 October), to minimize the effects of seasonal variation on evaluations of inter-annual changes in lake area. Images were corrected for Earth curvature and rotation, and the near-infrared band was utilized as the filter that best delineated shoreline boundaries^21^. Pixels were converted to ‘fuzzy classification' raster data. A total of 13 images were selected between 1986 and 2011, from which estimates of lake surface area were generated using ArcMAP v.10. Further methodological details can be found in the Supplementary Methods.

Overall regional lake surface area estimates derived from Landsat imagery were compared with major climate teleconnections including the El-Nino Southern Oscillation, Arctic Oscillation, North Atlantic Oscillation, Pacific Decadal Oscillation, North Pacific Index and the PNA pattern, as well as to global summer SSTA using the HadSST2 data set^22^. Regional lake surface area was also compared to the Adjusted and Homogenized Canadian Climate Data archive (publicly available from Environment and Climate Change Canada) for the nearby communities of Hay River and Yellowknife, and discharge records from the Trout and Cameron rivers (publicly available from Environment and Climate Change Canada), near Fort Simpson and Yellowknife, respectively. Individual Spearman's rank correlations were conducted (corrected for multiple comparisons using Bonferroni) to identify potential explanatory variables, which were related to reconstructed lake surface area based on Landsat imagery. Generalized additive models were generated for individual as well as multiple explanatory variables including all of the local climate and hydrological data as well as teleconnections. The Akaike information criterion was used to determine which model best explained the observed water level. All statistical analyses were carried out using the R statistical software environment^23^.

Sediment cores were collected from the centre of each of the five study lakes through the late-winter ice in March of 2012, using a Glew^24^ gravity corer (7.6 cm internal diameter). The sediment cores were extruded at 0.5 cm intervals using a Glew^25^ vertical extruder, and stored at <10 °C until further analysis. Select intervals from each core were dated with the ^210^Pb radiometric technique, using gamma spectrometry^26^ The constant rate of supply model^27^ was used for sediment age determination for all five sediment cores, which was applied with the program ScienTissiMe (Barry's Bay, Canada). The results for ^210^Pb dating can be found in Supplementary Fig. 3. Elemental analysis (organic carbon, nitrogen) and stable isotope analysis (δ^13^C, δ^15^N) in sediments was conducted following methods outlined in Korosi *et al*.^28^

A second sediment core was collected from Falaise Lake in late March 2013 for analysis of lignin-derived phenols. The core was sectioned into 0.5 cm intervals and shipped frozen back to the University of Ottawa. Sediment samples were ^210^Pb-dated using alpha spectrometry^26^ at the A.E. Lalonde AMS Facility (University of Ottawa) and a chronology was established using the constant rate of supply model (Supplementary Fig. 4). Laboratory analysis of lignin-derived phenols and elemental analysis followed methods outlined in Korosi *et al*.^28^ To ensure enough material was available for lignin analysis (which requires a total mass of 1.0 g freeze-dried sediment per interval), sediment intervals were grouped into 2.5 cm intervals.

### Data availability

Climate data are available from the Adjusted and Homogenized Canadian Climate Data Archive (Environment and Climate Change Canada: http://www.ec.gc.ca/dccha-ahccd/). River discharge data are available from the Environment and Climate Change Canada Wateroffice (https://wateroffice.ec.gc.ca/). Landsat 5 TM data are available from the United States Geological Survey (https://earthexplorer.usgs.gov/). All other relevant data are available from the authors.

## Additional information

**How to cite this article**: Korosi, J. B. *et al*. Broad-scale lake expansion and flooding inundates essential wood bison habitat. *Nat. Commun.* **8**, 14510 doi: 10.1038/ncomms14510 (2017).

**Publisher's note**: Springer Nature remains neutral with regard to jurisdictional claims in published maps and institutional affiliations.

## Supplementary Material

Supplementary Information Supplementary Figures 1-4, Supplementary Tables 1-3, Supplementary Notes 1-2, Supplementary Methods and Supplementary References
