# Supplementary material for: Broad-scale lake expansion and flooding inundates essential wood bison habitat
Source: Nat Commun. 2017 Feb 23;8:14510. doi: 10.1038/ncomms14510 (PMC5331212; doi:10.1038/ncomms14510)
Supplement: Supplementary Information — Supplementary Figures 1-4, Supplementary Tables 1-3, Supplementary Notes 1-2, Supplementary Methods and Supplementary References [file ncomms14510-s1.pdf]

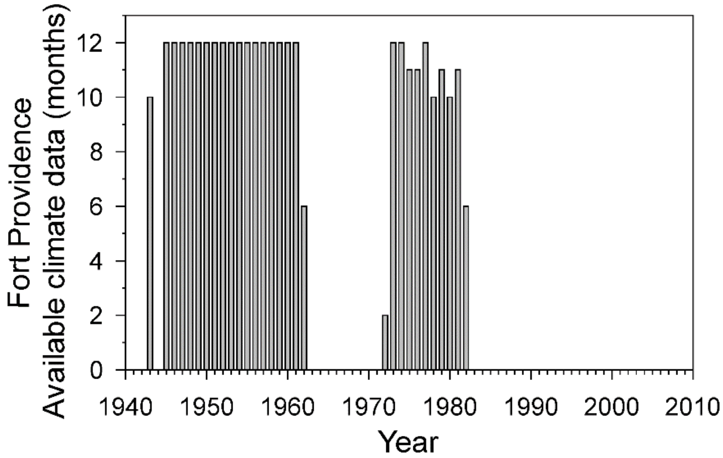

Supplementary Figure 1 –**Availability of climatic measurements for Fort Providence, NT.** Number of months for which climate records are available from Fort Providence, Northwest Territories, annually since 1940.

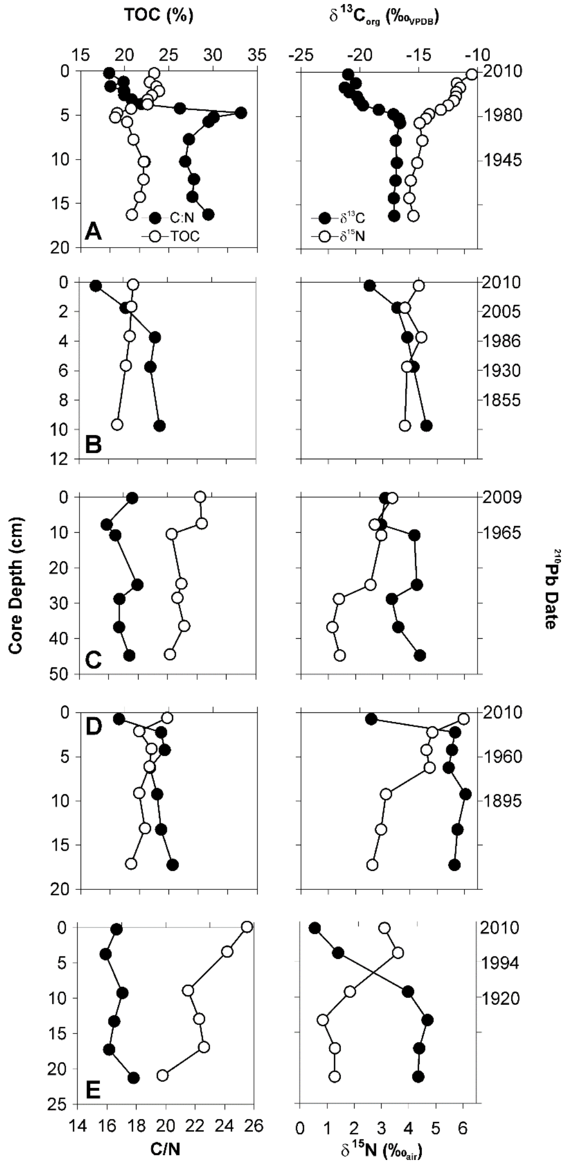

Supplementary Figure 2 – **Historical trends in geochemical proxies reconstructed from the dated sediment cores.** A) Falaise, B) Trio 1, C) Trio 3, D) Jackie, and E) Chan lakes, scaled by core depth. Sediment ages, derived from ^{210}Pb dating, are included as secondary axes. C/N – atomic organic carbon to nitrogen ratio; TOC – total organic carbon.

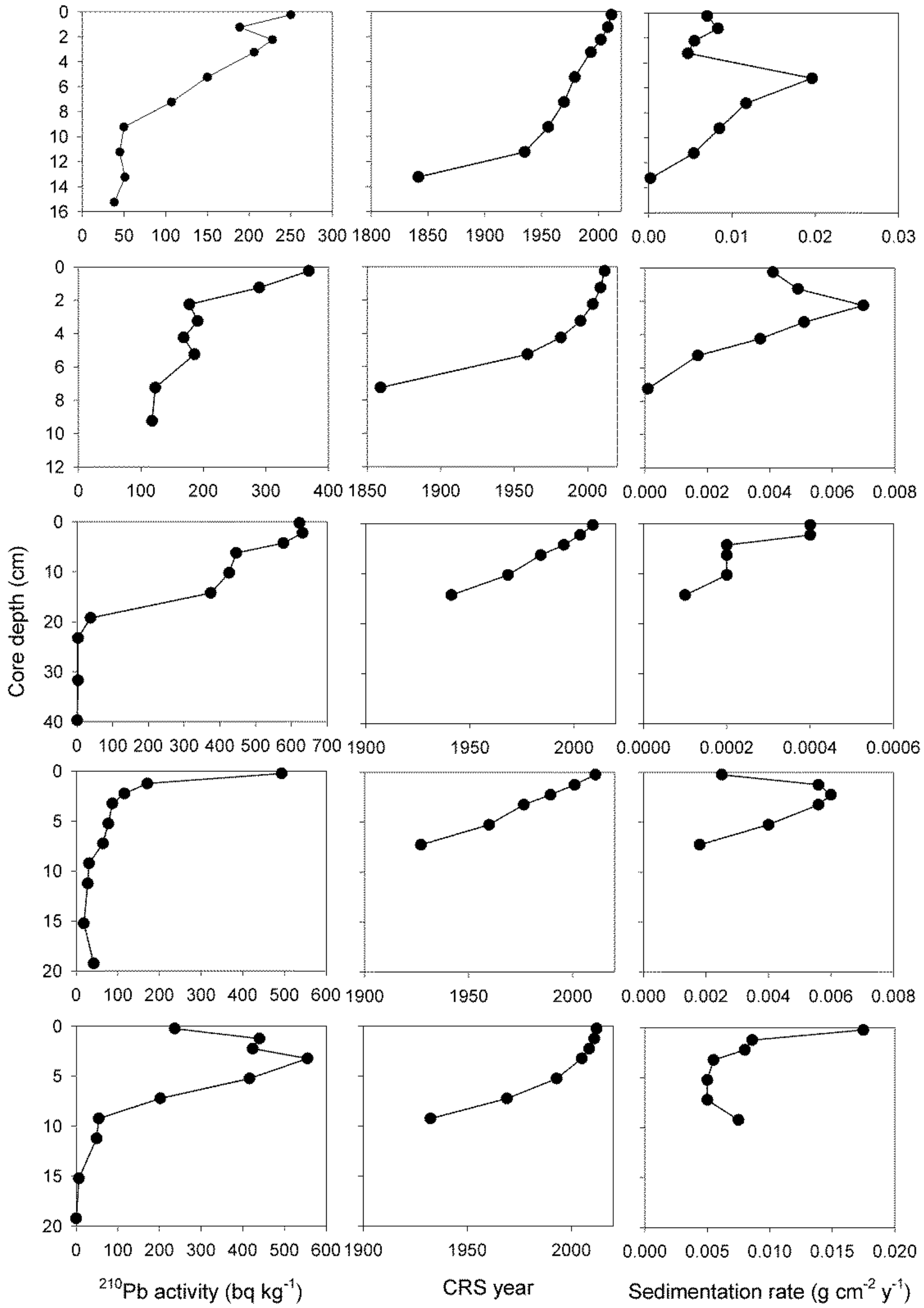

Supplementary Figure 3 – **Results for ^{210}Pb dating of sediment cores used in elemental and stable isotope analysis.** Core depth versus total ^{210}Pb radioisotopic activity; sediment core depth versus date based on the constant rate of supply model; sediment core depth versus inferred sedimentation rate for the five study lakes, in order from top to bottom: Falaise Lake (cored March, 2012), Trio 1 Lake, Trio 3 Lake, Jackie Lake, and Chan Lake.

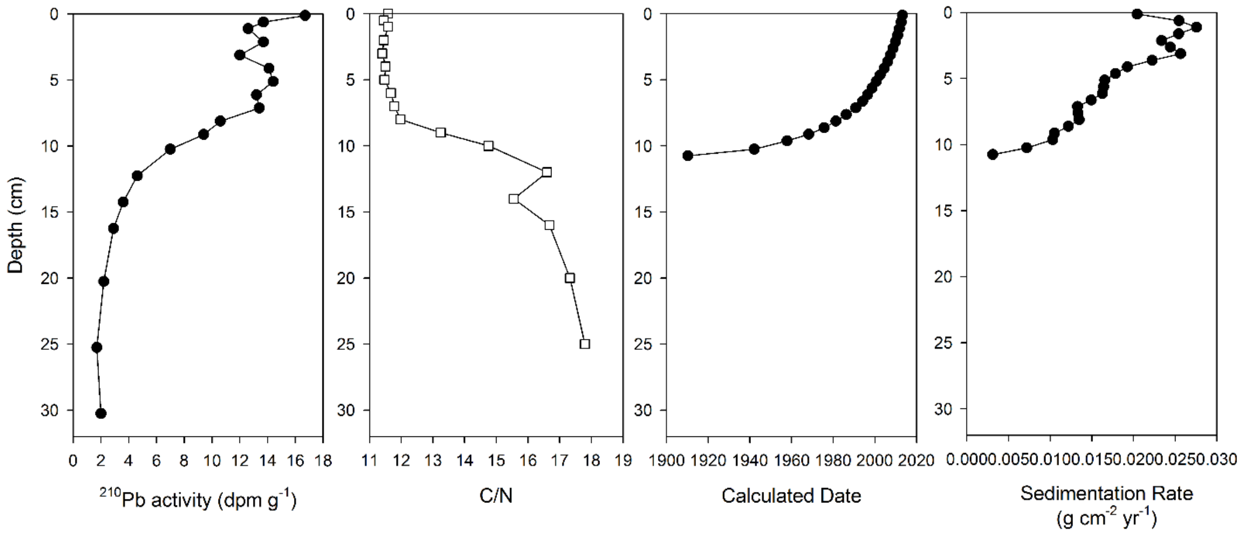

Supplementary Figure 4 - **Results for ^{210}Pb dating of the sediment core from Falaise Lake used for analysis of lignin-derived phenols.** Sediment core depth versus total ^{210}Pb radioisotopic activity; sediment core depth versus atomic organic carbon to nitrogen ratio (C/N); sediment core depth versus date based on the constant rate of supply model; sediment core depth versus inferred sedimentation rate. Radioisotopic activity and C/N were measured at 0.5 cm resolution. For analysis of lignin-derived phenols, sediments were combined into 2.5 cm intervals. Because of low ^{210}Pb activities, samples were counted on the spectrometer for between 114,000 to 362,000 seconds, as opposed to the standard practice of 82,000 for gamma spectroscopy. The slope of the resulting age-depth curve was variable, and the decrease in ^{210}Pb activity downcore is non-monotonic, with the upper (approximately) 7 cm exhibiting similar radioisotopic activity. This is likely the result of an influx of material following the rapid expansion of Falaise Lake, inundating terrestrial soils.

Supplementary Table 1 - **Geographic coordinates, 2010 surface area, and Landsat-derived percent change estimate since 1986 for the five lakes examined in this study.**

Lake	Latitude (°N)	Longitude (°W)	2010 Surface Area (ha)	Percent Change 1986-2010 (%)
Falaise	61.47642	116.15280	5637.6	+824
Trio 1	61.64026	116.05184	1036.4	+462
Trio 3	61.59762	116.07063	306.4	+20
Jackie	61.89678	116.55987	151.0	+313
Chan	61.89079	116.54170	66.2	+51

Supplementary Table 2 - **Spearman rank correlation analysis results comparing the climate record from Fort Providence with nearby Hay River and Yellowknife for seasonal and annual temperature and precipitation.**

Season	Station	Temperature	Precipitation
Annual	Hay River	**0.81 (<0.001)**	0.33 (0.13)
	Yellowknife	**0.85 (<0.001)**	**0.63 (<0.001)**
Winter	Hay River	**0.95 (<0.001)**	0.35 (0.08)
	Yellowknife	**0.96 (<0.001)**	0.26 (0.22)
Spring	Hay River	**0.95 (<0.001)**	**0.68 (0.002)**
	Yellowknife	**0.95 (<0.001)**	0.32 (0.09)
Summer	Hay River	**0.81 (<0.001)**	0.45 (0.02)
	Yellowknife	**0.85 (<0.001)**	**0.62 (<0.001)**
Fall	Hay River	**0.94 (<0.001)**	**0.62 (<0.001)**
	Yellowknife	**0.86 (<0.001)**	0.32 (0.11)

Correlation coefficients (r_s) are presented for each comparison with p values included in brackets. Comparisons that are significant at $\alpha=0.01$ (after correction for multiple comparisons) are in bold. The most significantly correlated record was used for further analyses.

Supplementary Table 3 – **Results of the generalized additive models**

Variable(s)	AIC	adjusted R^2
Cameron River + Trout River annual discharge	279.3	0.54
July to October Pacific / North American pattern	280.6	0.46
Annual temperature + Trout River annual discharge	281.1	0.47
Trout River annual discharge	281.7	0.42
Global summer sea surface temperature anomaly	284.2	0.29
Annual temperature + annual precipitation	286.7	0.19

Variables are ranked by increasing Akaike information criterion (AIC), and associated model adjusted regression coefficient.

Supplementary Note 1 – **Description of downcore trends for δ^{15}N or δ^{13}C in the five study lakes**

In addition to changes in C/N, an increase in δ^{15}N, and a decrease in δ^{13}C in cores from two of the expanding systems (Falaise and Jackie) were significantly correlated with increasing lake area. In Falaise Lake, δ^{15}N increased from ~4 to 6 ‰, while δ^{13}C decreased from -16‰ to -21‰ (Supplementary Figure 2). In Jackie Lake, a gradual increase in δ^{15}N began at ~8 cm (~1900), and increased again in ~2000, while δ^{13}C increased from -10 to -20 ‰ in the surface interval (Supplementary Figure 2). A study of long-term water level fluctuations in African Lake Victoria documented larger δ^{15}N values during wetter periods, which were attributed to a greater proportional use of dissolved inorganic nitrogen as a result of enhanced productivity from phosphorus release following flooding.[1] This represents a potential mechanism for the δ^{15}N changes observed in Falaise and Jackie lakes. The stable isotope record of Trio 1, however, was relatively complacent, and no changes in δ^{15}N were observed coincident with recent lake expansion. Although a gradual decrease in δ^{13}C was recorded in Trio 1, the timing did not correspond to lake level increases or changes in C/N (Supplementary Figure 2). Similarly, a

gradual increase in $\delta^{15}N$ and decrease in $\delta^{13}C$ was also recorded in Chan Lake above a core depth of 12 cm (around the turn of the century), with no corresponding change in C/N, or other indication of a lake expansion event (Supplementary Figure 2). This suggests that factors in addition to lake area change are also contributing to changes in these stable isotopes, and that C/N is a better indicator of lake area in these systems.

Supplementary Note 2 – **Discrepancy in the timing of the decrease in C/N between two duplicate sediment cores from Falaise Lake**

The timing of the decrease in C/N differed between the two duplicate sediment cores obtained from Falaise Lake. In the 2012 sediment core (core 1) used for elemental and isotopic analysis, the decrease in C/N occurred beginning in the mid-1980s, consistent with the recent period of flooding. In the 2013 sediment core (core 2), used for analysis of lignin-derived phenols, C/N decreased earlier, beginning in the 1940s, while the strong increase in cinnamyls indicative of increased soil organic matter inputs following flooding occurred consistent with the onset of lake expansion in the mid-1980s. C/N ratios are highly influenced by changes in algal production, especially in northern aquatic ecosystems with a short growing season. Climate warming can lead to an increase in algal production independent of lake expansion, due to a longer growing season and an increase in pelagic primary production, which in turn could lead to a decrease in C/N. This increase in primary production has been extensively documented in high-latitude regions.[2,3]

Analysis of sedimentary diatom assemblages (siliceous algae) in Falaise Lake showed an increase in the relative abundance of planktonic taxa and overall species diversity beginning in the 1940s, as well as increases in visual reflectance spectrally inferred chlorophyll *a* (J.

Thienpont, unpublished data), further suggesting that the earlier increase in C/N in core 2 may be related to the confounding effects of climate warming on autochthonous production. Overall C/N values in core 2 are lower than core 1, which may indicate that the location core 2 was taken from is integrating a greater proportion of sediment from the open-water environment, and consequently is more susceptible to subtle changes in algal production. Falaise Lake is a large, shallow lake (1-2 m depth), and even sediment cores taken in nearby locations may be subject to considerable variability. However, it is clear from both Falaise Lake sediment cores that the lowest C/N values were recorded in the most recent sediments. The uncertainty associated with the use of C/N as a proxy for organic matter sources was our primary motivation to strengthen our historical reconstruction of lake area changes using the lignin-derived phenol biomarker approach. The lignin-derived phenols, especially cinnamyls, show strong evidence of increasing allochthonous carbon inputs in recent decades, outside of the range we would expect based on general increases in terrestrial run-off that could be associated with climate warming, and no increases in lignin-derived phenols were observed in earlier sediment deposits spanning the last several centuries.

Supplementary Methods

Landsat 5 Thematic Mapper images were identified and utilized for all analyses, and accessed via the United States Geological Survey (USGS) Earth Explorer website (http://earthexplorer.usgs.gov/, last accessed Sept. 9, 2015). All LANDSAT files are corrected to Standard Terrain Correction Level 1T, using the Level 1 Product Generation System (Details at http://landsat.usgs.gov/Landsat_Processing_Details.php, last accessed Sept. 9, 2015). Images were selected from within a consistent time period, in order to minimize the effects of seasonal

variation on evaluations of inter-annual changes in lake area. The average maximum temperature in September (13.2°C) was selected as optimal, to minimize the influence of enhanced evaporation occurring during the hot summer months. The time period from August 12 to October 13th was selected as ideal for image selection, based on 30-year climate normalized data (1981-2010). No snow is present during this time period. Images with less than 10% cloud cover were selected, for a total of 13 images between the years 1986 and 2011. For images in which occasional clouds were still present, these small areas were masked from analysis using the Haze Removal tool, part of the Atmospheric Correction (ATCOR) wizard in PCI Geomatica 2013. Images were radiometrically calibrated using methods described in Finn et al.[4]

Images were classified into two broad categories: water and land. The near-infrared (NIR) band was used as the filter that best delineated shoreline boundaries, to avoid interference from flooded tree stands and floating vegetation.[5] Per-pixel, at-surface reflectance values were transformed to fuzzy membership values in ArcMap using the Fuzzy Membership tool. A Fuzzy Small transformation function was applied: low reflectance values (water) were given a membership of 1.0, high reflectance values (land) received a membership of 0.0, with all other values ranging between 1.0 and 0.0 membership. The resulting raster was then hardened (converted to crisp values) based on the membership of interest (three thresholds were examined for this project: 1.0, 0.5, and 0.1), producing a raster with only two categories: water or land. A minimum mapping unit (MMU) of 0.81 hectares (9 pixels) was implemented for this study.[6] A 3 x 3 pixel majority filter was implemented to generalize the image. Clustered pixels were grouped together using the Region Group tool in ArcMap. Clusters of eight pixels or less were re-classed as no-data and subsequently excluded from further analysis. The classified raster images were then converted to non-simplified vector shapefiles to calculate lake area.

Supplementary References

1. Talbot, M.R. & Laerdal, T. The Late Pleistocene-Holocene palaeolimnology of Lake Victoria, East Africa, based upon elemental and isotopic analyses of sedimentary organic matter. Journal of Paleolimnology 23, 141–164 (2000).

2. Michelutti, N., Wolfe, A.P., Vinebrooke, R.D., Rivard, B. & Briner, J.P. Recent primary production increases in Arctic lakes. Geophysical Research Letters 32: L19715, doi:10.1029/2005GL023693 (2005).

3. Thienpont, J.R., Rühland, K.M., Pisaric, M.F.J., Kokelj, S.V., Kimpe, L.E., Blais, J.M. & Smol, J.P. Biological responses to permafrost thaw slumping in Canadian Arctic lakes. Freshwater Biology 58: 337–353 (2013).

4. Finn, M.P., Reed, M.D. & Yamamoto, K.H. A Straight Forward Guide for Processing Radiance and Reflectance for EO-1 ALI, Landsat 5 TM, Landsat 7 ETM+, and ASTER. Unpublished Report from USGS/Center of Excellence for Geospatial Information Science. http://cegis.usgs.gov/soil_moisture/pdf/A%20Straight%20Forward%20guide%20for%20Processing%20Radiance%20and%20Reflectance_V_24Jul12.pdf (2012).

5. Van, T.T. & Binh, T.T. Application of remote sensing for shoreline change detection in Cuu Long estuary. Vietnam National University Journal of Science 25: 217-222 (2009).

6. Knight, J.F. & Lunetta, R.S. An experimental assessment of minimum mapping unit size. Geoscience and Remote Sensing 41: 2132-2134 (2003).
